# Phase separation dynamics of the *C. elegans* PGL-1 P granule protein in oocytes are sensitive to heat stress

**DOI:** 10.17912/micropub.biology.000476

**Published:** 2021-09-24

**Authors:** Brooklynne Watkins, Jennifer A. Schisa

**Affiliations:** 1 Central Michigan University

## Abstract

Phase separation has emerged as a widespread process of organizing the cytoplasm of diverse eukaryotic cells. In *C. elegans* oocytes, several RNA binding proteins are condensed into germ granules called P granules. Prior studies studying the phase transitions of RNA binding proteins in response to increased temperature have suggested that PGL-1 decondenses in oocytes in response to heat. Here, we confirm this finding with a new reporter strain and demonstrate the sensitivity of PGL-1 to temperature changes.

**Figure 1. PGL-1::GFP decondenses in oocytes in response to increased heat:**
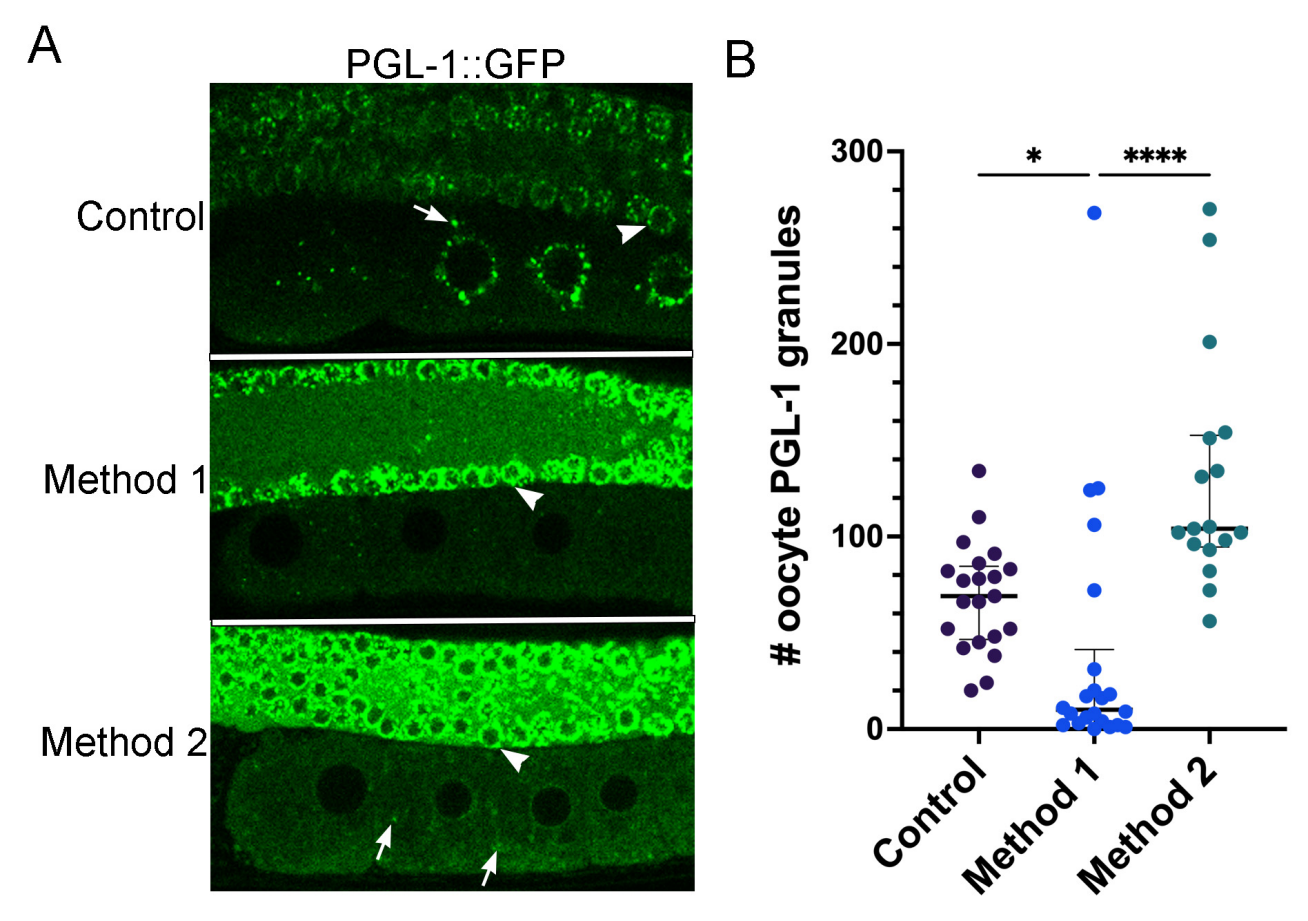
(A) In control, unstressed worms, PGL-1 is condensed into P granules in the proximal oocytes and distal nuclei. Method 1: Worms were incubated at 34°C for 40 min. in a microscope stagetop incubator and imaged at the same temperature. This method resulted in dispersal of PGL-1::GFP in the proximal oocytes of most worms. Method 2: Worms were incubated at 34°C for 40 min. in a standard incubator and imaged at room temperature. This method resulted in significantly more granules than Method 1; the granules were often detached from the nuclear envelope. Arrows indicate granules in oocytes; arrowheads indicate nuclear-associated granules in distal nuclei. (B) Graph shows the number of PGL-1::GFP granules in a single Z-slice of the -2 to -5 oocytes. Error bars represent median with interquartile range, *=P<0.05; ****=P<0.0001.

## Description

­In many cell types, subsets of RNA binding proteins adopt a default de-mixed state, condensing into ribonucleoprotein (RNP) granules (Alberti and Carra, 2018). In the *C. elegans* germ line PGL-1, an RGG domain protein, is condensed into P granules. P granules associate closely with nuclear pores of pachytene nuclei in the adult germ line but detach from nuclei as oocytes develop (Strome and Wood, 1982; Pitt *et al.*, 2000) and contribute to maternal mRNA regulation (Voronina, 2013). In contrast, other RNA binding proteins have a default mixed, or decondensed, state in the adult germ line. For example, the KH domain protein MEX-3 is largely diffuse throughout the cytosol of oocytes and condenses into P granules in early embryos (Draper *et al.*, 1996).

Multiple studies have shown that several types of RNA binding proteins undergo dynamic phase separation during certain environmental stresses and extended meiotic arrest. P-granule proteins, such as PGL-1, P-body proteins such as CGH-1, and stress granule proteins such as TIAR-2 are all detected in large RNP granules in meiotically arrested oocytes (Jud *et al.*, 2008; Noble *et al.*, 2008; Wood *et al.*, 2016). The RNP granules are hypothesized to maintain oocyte quality during delays in fertilization. Similarly, in a study of heat stress responses, P-body and stress granule proteins also condense into large RNP granules in oocytes (Jud *et al.*, 2008). In contrast, in the same study a summary table states that PGL-1 granules are not detected after heat stress; however, images of this experiment were not published (Jud *et al.*, 2008).

Recent studies using the PGL-1::GFP reporter strain demonstrate that PGL-1 decondenses rapidly in the early embryo after the temperature is upshifted to 34°C (Putnam *et al.*, 2019). P granules in the early embryo are similar, but not identical to P granules in oocytes. As mentioned above, a subset of proteins are transient P granule components. Since MEG-3 plays an important role in stabilizing PGL-1 granules in the early embryo, but does not associate strongly with P granules prior to fertilization, it is not necessarily the case that P granule dynamics in oocytes will recapitulate dynamics observed in embryos (Wang *et al.*, 2014). The prior heat stress study by Jud *et al.*, was conducted before CRISPR reporter strains were available; therefore, we asked if PGL-1::GFP decondenses in oocytes in response to increased temperature. PGL-1 was detected in perinuclear and cytoplasmic P granules as expected in control oocytes (median of 69 granules) and in the distal germ nuclei. In contrast, after 40 minutes at 34°C in a stagetop incubator (Method 1), in the majority of worms PGL-1 appeared dispersed, with significantly fewer granules detected in the proximal -2 to -5 oocytes (median of 10 granules) (Figure 1). This result appears similar to the original result described, but not shown, that detected PGL-1 by immunofluorescence after fixing worms immediately after being exposed to 34°C via a standard incubator (Jud *et al.*, 2008). As a side note, we did not detect similar dispersal of PGL-1 in the distal nuclei, suggesting nuclear-associated PGL-1 granules may be less sensitive to temperature changes (arrowheads, Figure 1). While this manuscript was in revision, an independent study found similar results in oocytes and distal nuclei (Fritsch *et al.*, 2021). Interestingly, when we incubated the PGL-1:GFP worms in a standard incubator and imaged without a stagetop incubator (Method 2), many more PGL-1 granules were detected in oocytes than with Method 1 (median of 104 granules; Figure 1). This may not seem surprising since the worms could be expected to recover at ambient temperature, and many phase transitions are reversible. However, it is notable, as a practical consideration, that the phase separation of PGL-1 into granules occurred quickly, within 15 minutes at ambient temperature.

From these results with the PGL-1::GFP strain, we confirm that in proximal oocytes PGL-1 is extremely sensitive to temperature changes, and behaves as a dynamic, liquid-like protein, similar to its behavior in the early embryo (Jud *et al.*, 2008; Putnam *et al.*, 2019). While short fluctuations in temperature during heat stress experiments may not adversely affect all fluorescent reporter strains, they can clearly affect phase transitions of liquid-like proteins such as PGL-1.

## Methods

**Heat Stress**

*Method 1.* The Tokai Hit stage top incubator was used at 34°C with the Nikon A1R laser scanning confocal microscope. Young adult PGL-1::GFP worms were placed on small NGM plates and incubated in the stage top incubator for 40 minutes at 34°C. Within two minutes at room temperature, the worms were transferred to a 2% agarose pad prior to imaging at 34°C. The temperature remained at 34°C throughout imaging, and imaging was completed within 15 minutes.

*Method 2.* NGM plates with young adult PGL-1::GFP worms were placed in a standard incubator at 34°C for 40 minutes. Worms were transferred to a 2% agarose pad and imaged at ambient temperature on the Nikon A1R confocal microscope. Worms were at ambient temperature for 8-15 minutes before images were acquired.

**Microscopy**

Worms were mounted on a 2% agarose pad and imaged in 6.25mM levamisole to immobilize worms, or 2.5mM levamisole to prevent bursting of heat-stressed worms. Imaging was performed using a Nikon A1R confocal microscope using a 60x N.A. 1.2 water-immersion objective. 0.5mM Z-slices were collected. Images were pseudo-colored using Adobe Photoshop.

**Statistical Analyses**

ImageJ was used to determine the number of granules in the mid-focal Z-slices of the most proximal (-2 to -5) oocytes. An ANOVA/ Kruskal Wallis test was conducted using GraphPad Prism v.8 to analyze statistical significance. GPower 3.1 was used to conduct a power analysis to determine the sample size.

## Reagents

**Table:** 

Strain	Genotype	Available from
JH3269	*pgl-1(ax3122[pgl-1::gfp]) IV*	CGC
